# An identical-by-descent segment harbors a 12-bp insertion determining fruit softening during domestication and speciation in *Pyrus*

**DOI:** 10.1186/s12915-022-01409-w

**Published:** 2022-10-01

**Authors:** Bobo Song, Xiaolong Li, Beibei Cao, Mingyue Zhang, Schuyler S. Korban, Li’ang Yu, Wenxi Yang, Kejiao Zhao, Jiaming Li, Jun Wu

**Affiliations:** 1 College of Horticulture, State Key Laboratory of Crop Genetics and Germplasm Enhancement, Nanjing Agricultural University, Nanjing, 210095 China; 2 Present Address: Key Laboratory of Quality and Safety Control for Subtropical Fruit and Vegetable, Ministry of Agriculture and Rural Affairs, College of Horticulture Science, Zhejiang Agriculture and Forestry University, Hangzhou, 311300 China; 3 Department of Natural Resources and Environmental Sciences, University of Illinois at Urbana-Champaign, Urbana, IL 61801 USA; 4 The Boyce Thompson Institute, Cornell University, Ithaca, NY 14850 USA; 5 Department of Food Science and Human Nutrition, University of Illinois at Urbana-Champaign, Urbana, IL 61801 USA

**Keywords:** Pear, Domestication, IBD, *TIC55*, Tandem duplication, Fruit softening

## Abstract

**Background:**

Although the wild relatives of pear originated in southwest China, this fruit crop was independently domesticated and improved in Asia and Europe, and there are major phenotypic differences (e.g., maturity and fruit firmness) between Asian and European pears.

**Results:**

In this study, we examined the genomes of 113 diverse pear accessions using an identity-by-descent (IBD) approach to investigate how historical gene flow has shaped fruit firmness traits in Asian and European pears. We found a 3-Mbp IBD-enriched region (IBD-ER) that has undergone “convergent domestication” in both the Asian and European pear lineages, and a genome-wide association study (GWAS) of fruit firmness phenotypes strongly implicated the *TRANSLOCON AT THE INNER CHLOROPLAST ENVELOPE55* (*TIC55*) locus within this 3-Mbp IBD-ER. Furthermore, we identified a tandem duplication that includes a 12-bp insertion located in the first exon of *TIC55* that is uniquely present in Asian pears, and expression analysis showed that the pear *TIC55* gene is highly expressed in Asian pear, while it is weakly or not expressed in European pear; this could contribute to the differences in fruit firmness between Asian and European pear fruits.

**Conclusions:**

Our findings provide insights into how pear fruit softening has been impacted during domestication, and we identified candidate genes associated with fruit softening that can contribute to the breeding and improvement of pear and other fruit crops.

**Supplementary Information:**

The online version contains supplementary material available at 10.1186/s12915-022-01409-w.

## Background

Pears (species in the genus *Pyrus* L.), which belong to the subfamily Pomoideae in the botanical family Rosaceae, comprise the third most important fruit crop in the world, ranking just after apple with a yield of ~ 16.1 million tons (2020, FAOSTAT) per year [1]. There are at least 22 widely recognized pear species [2], and more than 5000 pear accessions are currently distributed worldwide [3, 4]. In 2018, a pear domestication model proposed that the pear originated in southwest China, was disseminated through central Asia, and eventually underwent independent domestication in the Asian and European pear populations [3].

Beneficial reciprocal gene flow via interspecific hybridization is an important way to improve phenotypic traits during domestication [5, 6]. With the release of more genomic DNA resequencing data, gene flow has been widely investigated in many plant species. In rice, introgression analysis has determined that most rice types exhibit distinct degrees of admixture, with many accessions within a population conserving particular identity-by-descent (IBD) tracts due to artificial selection [7]. In maize, long conserved IBD tracts have been detected between tropical and temperate inbred lines, which may be attributed to gene flow between tropical and temperate maize that must have occurred during domestication and genetic improvement [8]. Pear, a typical gametophytic self-incompatible plant, is an obligate out-crosser [9]. Therefore, it is expected that there is a more likely gene flow between wild and cultivated pear populations. During domestication, pears underwent dramatic morphological and physiological changes, and these differences are considered to be viable candidate targets for investigating pear domestication and improvement.

**Fig. 1:**
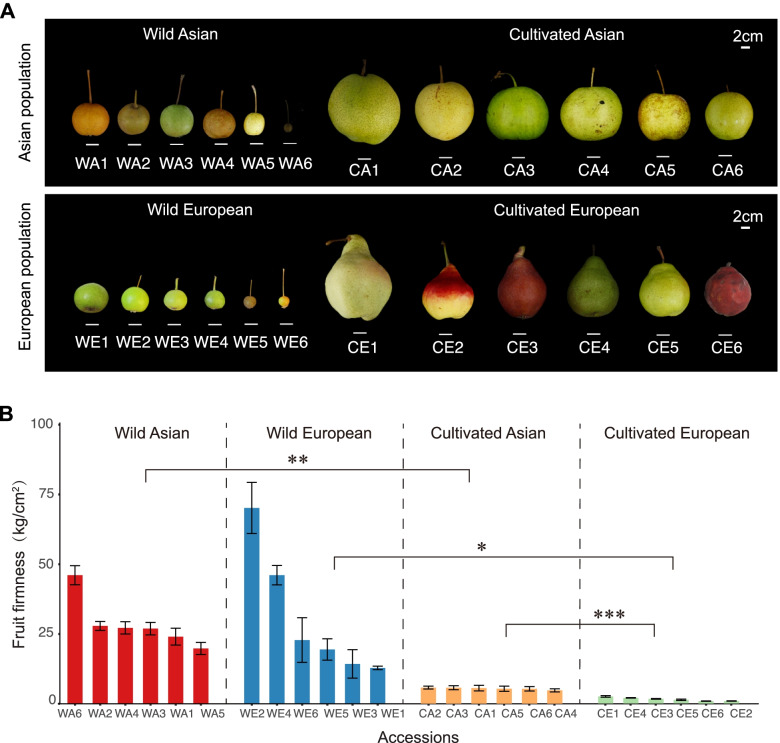
Phenotypes of pear fruits and fruit firmness in the four pear populations. **A** Mature fruits of wild Asian (WA), wild European (WE), cultivated Asian (CA), and cultivated European (CE) pears. **B** Firmness of WA, WE, CA, and CE fruits (Additional file 1: Table S1). The mean separations were conducted using the *T*-test in the R language. The asterisks indicate significant differences in fruit firmness between the populations (*p < 0.05, **p < 0.01, and ***p < 0.001)

Firmness is one of the most important fruit traits because it determines the shelf-life and commercial value of the fruit. Compared with wild pears, the firmness of cultivated pear fruits is markedly lower; moreover, the firmness of cultivated Asian pear fruits is higher than that of cultivated European pears (Fig. 1A, B; Additional file 1: Table S1). This suggests that fruit firmness is likely to be one of the typical phenotypic traits that must have been involved in the independent domestication of Asian and European pears. Fruit softening is closely related to the degradation of cell walls that are mainly composed of pectin, cellulose, and hemicellulose [10]. The degradation of cell walls involves the interaction of many cell wall-related enzymes, including polygalacturonase (PG) [11], pectin methylesterase (PME) [12], *β*-galactosidase (*β*-GAL) [13], and pectate lyases (PLs) [14]. Ethylene (ET) is the main hormone that promotes fruit ripening and senescence, and it plays an important role in the fruit softening process. Therefore, ET biosynthesis and signal transduction are important factors that affect fruit softening. For example, the *ACS1* and *ACO1* genes have been shown to regulate ET biosynthesis in apple fruit during ripening [15, 16]. During the signal transduction process, ethylene response factor (ERF) family members are widely reported to be involved in regulating downstream ET-responsive genes, such as *SR1* [17], *LeERF2* [18], *MaERF9* and *MaERF11* [19], and *ERF4* [20]. *SR1*, a member of the tomato *CALMODULIN BINDING TRANSCRIPTION ACTIVATOR* (*CAMTA*) family, is involved in ET signal transduction by regulating the expression level of *ETHYLENE INSENSITIVE3* (*EIN3*) [17]. In addition, fruit softening is one of the important main characteristics of senescence, and genes involved in plant senescence have been widely studied. In *Arabidopsis*, knocking out the expression of *CAMTA3*, a gene that encodes a calmodulin-binding protein, contributes to early senescence [17]. Moreover, it has been reported that the *TRANSLOCON AT THE INNER CHLOROPLAST ENVELOPE55* (*TIC55*) gene plays a vital role in chlorophyll breakdown and is associated with a translocon for proteins via an inner chloroplast membrane [21].

In this study, resequencing data from 113 pear accessions was analyzed to explore the potential role(s) of gene flow and the phenotypic effect(s) on fruit firmness during the domestication of pear (Additional file 2: Table S2). Our findings included (1) a landscape of the incidence of gene flow in the genomes of Asian and European populations and (2) fruit firmness-related phenotypic effects resulting from gene flow during domestication must have accompanied the divergence of the Asian and European pear lineages.

## Results

### Genome-wide identification of putative identity-by-descent (IBD) tracts in 113 *Pyrus* accessions

**Fig. 2:**
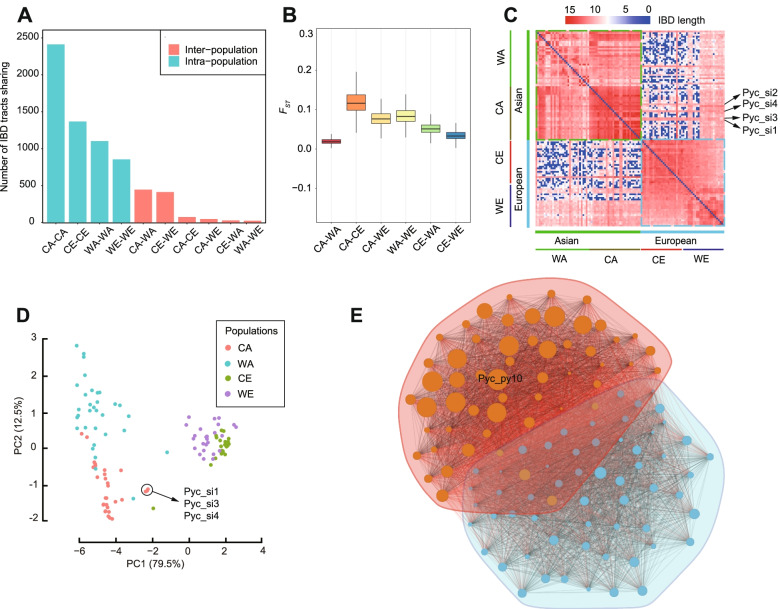
Characterization of putative IBD tracts in the genomes of the 113 *Pyrus* accessions. **A** The number of putative IBD tracts is based on the average pairwise individual intra-population and inter-population comparisons. The blue bars indicate intra-population comparisons, while the red bars indicate inter-population comparisons. **B** Boxplot of *F*_*ST*_ values for pairwise comparisons between the two different populations. **C** Heatmap showing the IBD tract sharing (length) among the 113 pear accessions. The 113 pear accessions comprised wild Asian (green line), wild European (blue line), cultivated Asian (brown line), and cultivated European (red line) pears. The average length of the IBD tracts within a single accession was denoted as zero in this study. The color scale at the top indicates the average lengths of IBD tracts between any two different accessions. **D** Principal component analysis (PCA) based on the IBD tract data. Red dots correspond to cultivated Asian pears (CA), green dots to cultivated European pears (CE), light blue dots to wild Asian pears (WA), and purple dots to wild European pears (WE). **E** IBD sharing among the 113 pear accessions. Dots correspond to the accessions, while lines connecting any two dots represent IBD tract sharing. The dot sizes correspond to the number of IBD tracts shared

In our study, we used genome sequencing data from 113 pear accessions collected from previous research; the accessions came from four pear populations and included 31 cultivated Asian, 32 wild Asian, 25 cultivated European, and 25 wild European accessions. We cataloged IBD tracts at the whole-genome level to explore the gene flow within/across these four populations. A total of 3,320,796 IBD tracts were identified in pairwise comparisons of the 113 pear accessions. We divided the identified IBD tracts from all 10 pairwise comparisons into two categories: intra-population tracts and inter-population tracts. Among the intra-population tracts, the cultivated Asian-cultivated Asian (CA-CA) comparison showed the highest extent of IBD sharing (2411), and the wild European-wild European (WE-WE) comparison showed the lowest extent of IBD sharing (1367). These results suggest that the cultivated Asian pear population has experienced the highest extent of intra-population genetic exchange among the four populations. For the inter-population comparisons, we found that cultivated Asian and wild Asian showed slightly higher levels of IBD sharing compared to that between cultivated European and wild European pears (Fig. 2A), indicating a relatively higher level of gene flow between wild Asian and cultivated Asian pears. Notably, the IBD tract data corresponds with our finding from an *F*_*ST*_ analysis showing that the degree of divergence in cultivated Asian and wild Asian pear (*F*_*ST*_ = 0.021) is reduced compared to cultivated European and wild European pear (*F*_*ST*_ = 0.035) (Fig. 2B).

We also estimated the length distribution of the IBD tracts. Most IBD tracts had lengths between 1500 and 3500 bp in all 10 pairwise comparisons (Additional file 3: Table S3 and Additional file 14: Fig. S1). The number of IBD tracts decreased sharply with increasing tract length for both the intra-population and inter-population comparisons; this can likely be explained by the fact that longer IBD tracts are more likely to be disrupted by genetic recombination [6, 22]. A heatmap and principal component analysis (PCA) based on the IBD tract data showed that the 113 pear accessions were divided into two groups that corresponded to Asian and European populations (Fig. 2C, D). Except for three *Pyrus sinkiangensis* and a single *Pyrus armeniacaefolia* accessions, Asian pears in this PCA model were distinct from the European pears; this separation likely reflects the long-term geographical separation of Asian and European pears.

To investigate which accessions have contributed the highest proportion of genetic background to the pear gene pool, we generated an IBD sharing network and used it to explore the relationships among the pear accessions. This is shown in Fig. 2E, where the dot sizes correspond to the number(s) of shared IBD tracts. We found that the *Pyc_py10* (“Wasekozo”) accession of *Pyrus pyrifolia* contained the highest level of IBD sharing (140,546), reflecting the known fact that “Wasekozo” has been widely used as a hybrid parent in Asian pear breeding programs [23]. For example, the Japanese pear variety “Kosui,” one of the most widely grown Asian pear cultivars, was derived from a cross between “Wasekozo” and “Kikusui” (MAFF, https://www.maff.go.jp/j/tokei/index.html).

### Identification of IBD-enriched chromosomal regions

**Fig. 3:**
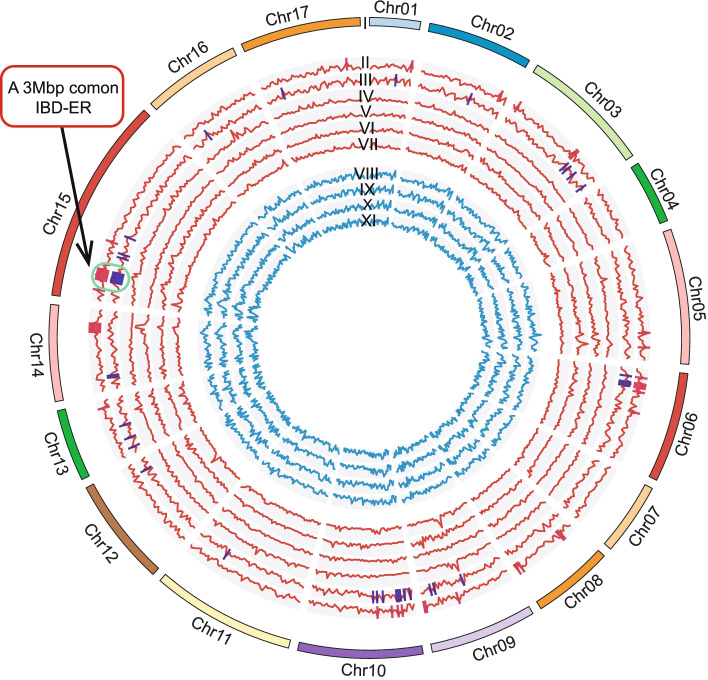
Circos diagram showing the frequency of IBD tracts on the 17 pear chromosomes in 500-kb windows. The rings in the diagram, from outer to inner, represent the chromosomes, and these include the following: (I) chromosomes and (II–VII red) chromosomal distribution of IBD tracts in comparison with the different populations; IBD-ERs in European and Asian pears are highlighted in red and blue, respectively. (II) Cultivated European-wild European; red bars indicate IBD-enriched regions (IBD-ERs, the number of IBD tracts in the top 5% of regions). (III) Cultivated Asian-wild Asian; blue bars indicate IBD-enriched regions (IBD-ERs, the number of IBD tracts in the top 5% of regions). (IV) Wild Asian-wild European. (V) Cultivated Asian-wild European. (VI) Cultivated European-wild Asian. (VII) Cultivated Asian-wild European. (VIII–XI blue) Chromosomal distribution of IBD tracts within the four pear populations: (VIII) cultivated European-cultivated European, (IX) cultivated Asian-cultivated Asian, (X) wild Asian-wild Asian, and (XI) wild European-wild European pears

We also measured the number of IBD tracts in 500-kb windows across the 17 pear chromosomes (Additional file 4: Table S4). Extensive but uneven distributions of IBD tracts were identified in all the 10 pairwise comparisons (Fig. 3). At the chromosome level, the fewest IBD tracts were detected for chromosome 4 (2.87%) in the comparison between cultivated Asian and wild Asian (CA-WA) pears, and the fewest IBD tracts from the cultivated European-wild European (CE-WE) pear comparison were for chromosome 7 (3.09%). It was also interesting to note that chromosome 15 had the most significant number of IBD tracts for both the CA-WA (12.52%) and CE-WE (11.99%) comparisons (Additional file 15: Fig. S2A and Fig. S2B).

We next defined the top 5% of windows (39) with the most significant number of IBD segments as IBD-enriched regions (IBD-ERs) (Additional file 5: Table S5). In the CA-WA comparison, a total of 1725 genes were identified in IBD-ERs (Asian IBD-ERs), while a slightly lower number of genes (1354) was identified within IBD-ERs in the CE-WE comparisons (European IBD-ERs) (Additional file 6: Table S6 and Additional file 7: Table S7). A Gene Ontology (GO) term analysis revealed that genes with functional annotations related to multiple aspects of carbohydrate metabolism were enriched in the Asian pear IBD-ERs (*p*-value < 0.05, Additional file 15: Fig. S2C and Fig. S2D). Of particular note is a 3-Mbp region on chromosome 15 containing 446 protein-encoding genes that was detected as a common IBD-ER in both the Asian and European pear populations (Fig. 3). Notably, a previous study reported a QTL (*MEST050*) related to harvest time that overlaps this 3-Mbp region [24], and a KEGG pathway analysis we conducted indicated that genes in this region were enriched for annotated functions related to fruit ripening (e.g., “Zeatin biosynthesis”) (Additional file 16: Fig. S3). These findings suggest that fruit maturity-related traits have been of particular interest to humans and were selected during the domestication processes for both Asian and European pears.

### A 3-Mbp IBD-ER shared between Asian and European populations experienced positive selection during pear domestication

**Fig. 4:**
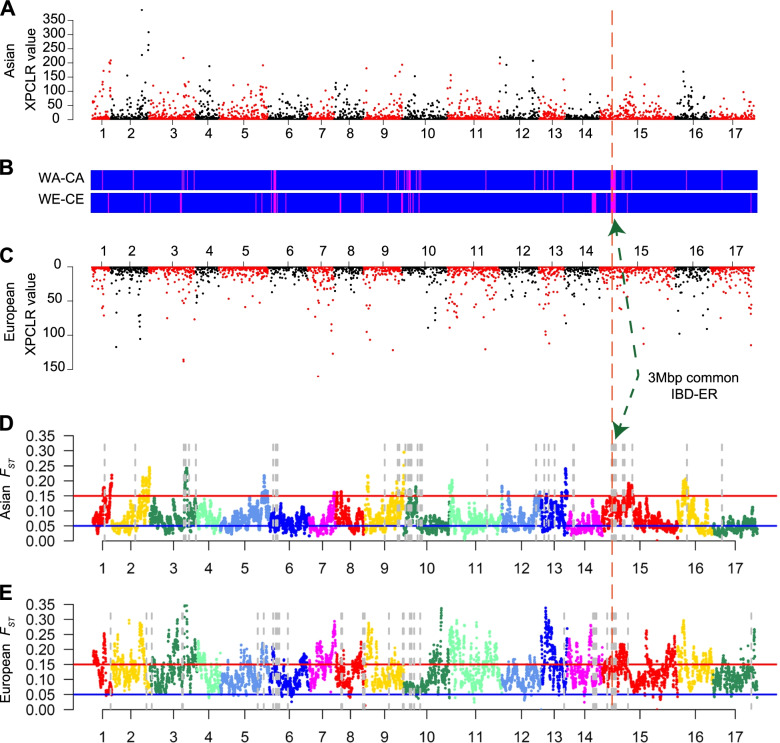
Selection signals of IBD-enriched regions (IBD-ERs) in Asian and European pear populations. XP-CLR values of whole genomes between wild and cultivated populations of Asian (**A**) and European (**C**) pears. **B** Distribution of IBD-ERs. The magenta regions indicate the IBD-ERs, while the blue regions indicate non-IBD-ERs. Distribution of pairwise *F*_*ST*_ values on the 17 chromosomes between wild and cultivated Asian pears (**D**) and European pears (**E**). Gray dotted lines indicate the IBD-enriched regions. The 3-Mbp IBD-enriched region (IBD-ER) is indicated by a green dashed line and arrowheads in **B** and **D**

We used two methods to identify selective sweeps among the IBD-ERs to explore which, if any, have undergone artificial selection. First, we performed a cross-population likelihood ratio analysis of the whole genomes between wild and cultivated populations using the XP-CLR program [25]. We considered regions harboring XP-CLR values in the top 20% (Asian: XP-CLR value ≥ 5.17; European, XP-CLR value ≥ 2.33) as putative domestication regions exhibiting selection signals (Fig. 4A–C) [26]. Approximately 17.9% and 20.5% of the IBD-ERs showed signals of positive selection among Asian and European pears, respectively. It is notable that only two IBD-ERs (500-kb windows; Chr15: 6,000,001–6,500,000, Chr15: 7,500,001–8,000,000) displayed selection signatures in both the Asian and European populations, and these two selection signals were both within the aforementioned 3-Mbp IBD-ER on chromosome 15 (Chr15: 5,000,000–8,000,000). This finding supports our earlier conclusion that the 3-Mbp IBD-ER did indeed undergo positive selection during the domestication processes for both Asian and European pears.

Second, we identified the population differentiation level (*F*_*ST*_) at the whole-genome level between wild and cultivated populations using the VCFtools program. Briefly, *F*_*ST*_ values for a single genomic region between 0.05 and 0.15 indicate that two populations have a moderate degree of genetic differentiation; an *F*_*ST*_ value > 0.15 suggests that two populations are separated. Our analysis showed that 17.9% and 15.4% of the IBD-ERs had *F*_*ST*_ values > 0.15 in the Asian and European populations, respectively (Fig. 4D, E). Conspicuously, the only IBD-ER (Chr15: 7,500,001–8,000,000) that had an *F*_*ST*_ value > 0.15 in both the Asian and European populations was located within the 3-Mbp IBD-ER on chromosome 15. Thus, both of the methods we used to evaluate selective sweeps support the conclusion that the 3-Mbp IBD-ER on chromosome 15 has experienced strong selection. In addition, cross-population extended haplotype homozygosity XP-EHH analysis also supported this conclusion. There were a total of 448 and 91 selection signals in the 3-Mb IBD-ER in the wild Asian-vs-cultivated Asian (WA-CA) and wild European-vs-cultivated European (WE-CE) comparisons, respectively (Additional file 17: Fig. S4). The detection of positive selection for this common region in the two independently domesticated pear populations suggests that we have detected an example of “convergent domestication” in our study [27, 28]; this is apparently similar to the previously reported case in *Brassica* where 15 convergent selection loci were identified during the independent domestications of *Brassica rapa* and *Brassica oleracea* [29].

### GWAS for fruit firmness identifies candidate causal genes within the 3-Mbp IBD-ER shared by Asian and European pears

**Fig. 5:**
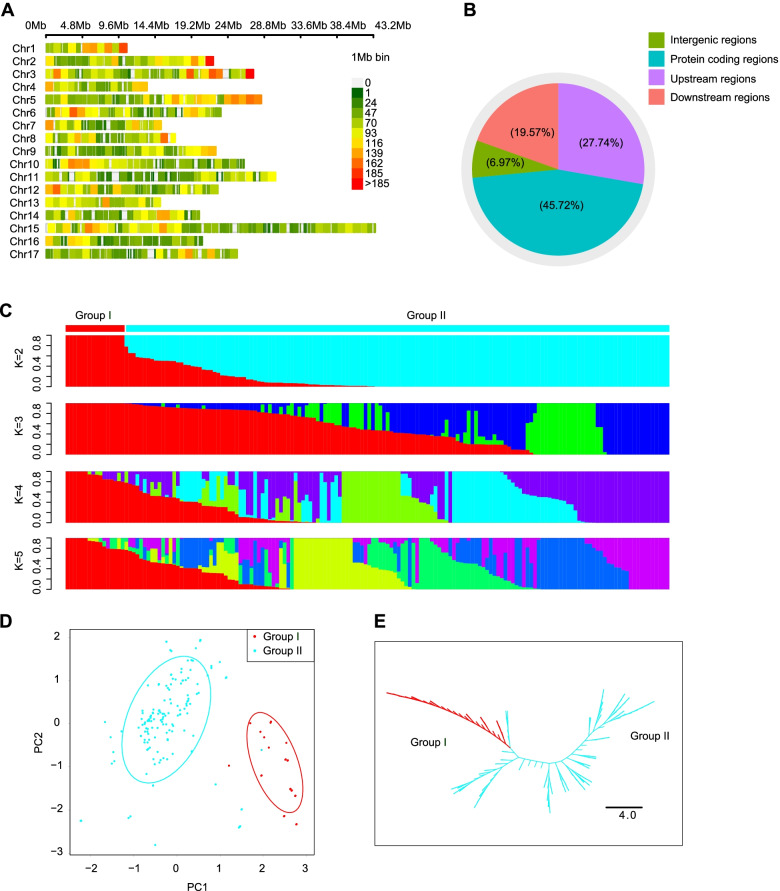
Genomic variation and population structure in the 164 pear accessions used for genome-wide association analysis. **A** Distribution of the positions of the 29,269 high-quality SNPs used in the GWAS on the 17 pear chromosomes. The color scale to the right indicates the numbers of SNPs within a 1-Mb window. Red indicates a high density of SNPs within a 1-Mb window, while green indicates a low density of SNPs within a 1-Mb window. **B** The percentages of SNPs that mapped to four different genomic regions, including intergenic regions, protein-coding regions, upstream regions, and downstream regions. **C** Population structure analysis of the 164 pear accessions at *K* = 2–5. **D** PCA analysis of the 164 pear accessions. **E** Unrooted neighbor-joining tree of the 164 pear accessions. Group I (mostly European pears) is shown in light blue, and group II (mostly Asian pears) is shown in red

We also used the 200 K AXIOM® PyrSNP array to genotype a germplasm diversity panel specifically assembled for cultivated pears (comprising 164 pear cultivars) that we used in a GWAS examining fruit traits (Additional file 8: Table S8). Very briefly, 29,269 high-quality SNPs were retained after filtering (Fig. 5A and Additional file 9: Table S9 and Additional file 10: Table S10), among which 3566 were both positioned within protein-coding regions and resulted in predicted changes in amino acids or altered start/stop properties for transcription (Fig. 5B and Additional file 11: Table S11). Population structure, PCA, and neighbor-joining phylogenetic tree analyses all supported dividing these 164 cultivated pear accessions into two groups, with group II comprising mostly Asian pears and group I comprising mostly European pears (Fig. 5C–E). Of note, and consistent with both results from our analysis of the four pear populations and conclusions from a previous study [3], nucleotide diversity (*π*) and linkage disequilibrium (LD) analysis strongly support the notion that Asian pears are substantially more genetically diverse than European pears.

**Fig. 6:**
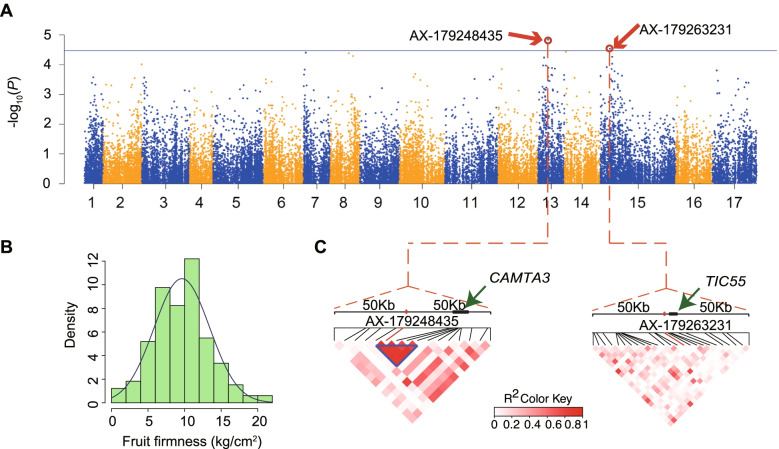
A genome-wide association study (GWAS) for fruit firmness. **A** Results of GWAS for fruit firmness determined using the GAPIT package in R. A blue line indicates a significant threshold (3.41 × 10^−5^), while a red circle indicates a SNP marker that is significantly associated with fruit firmness. **B** A histogram showing the distribution of fruit firmness in the 164 pear accessions included in the GWAS population. **C** Two LD heatmaps of the chromosomal regions 50-kb upstream and downstream of the two significant SNPs AX-179248435 and AX-179263231

Before conducting GWAS, phenotypic data for fruit firmness was collected for all 164 accessions from the cultivated pear diversity panel analyzed with the 200 K AXIOM® PyrSNP array. The fruit firmness data demonstrated a normal distribution (Shapiro–Wilk normality test, *W* = 0.988, *p*-value = 0.180) (Fig. 6A, B) and was used for genome-wide association analysis based on a mixed linear model (MLM). The GWAS detected two loci, AX-179248435 on chromosome 13 and AX-179263231 on chromosome 15, that were significantly associated with fruit firmness (Fig. 6C and Additional file 18: Fig. S5). Based on a previous genome-wide association study in pear, we used 100-kb windows to detect candidate genes [30]. A total of 32 candidate genes were identified from the flanking regions of the two fruit firmness-associated sites: 12 genes from the site on chromosome 13 and 20 genes on chromosome 15 (Additional file 12: Table S12).

**Fig. 7:**
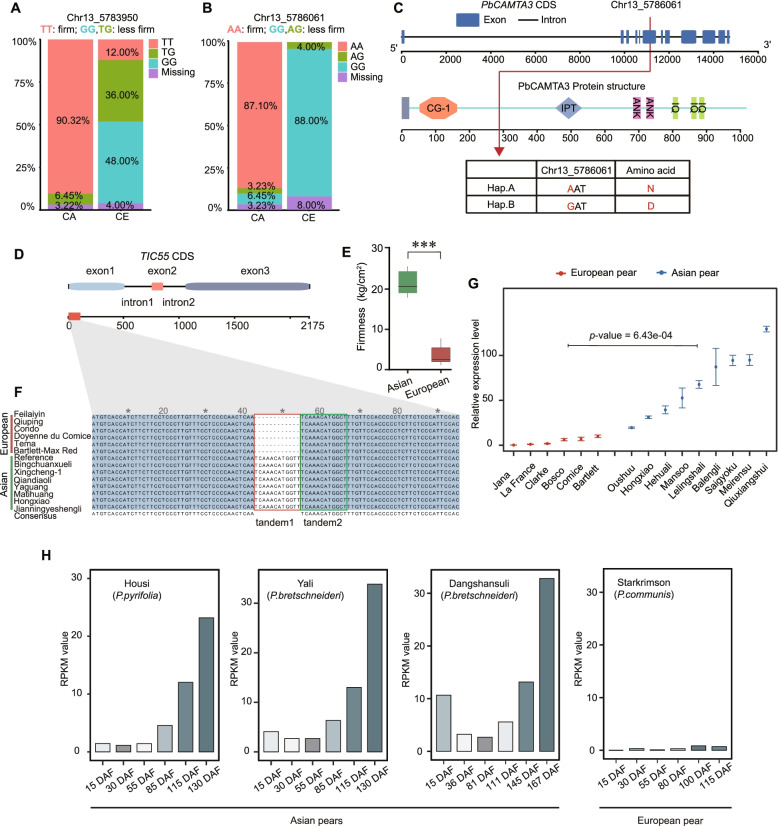
The genetic divergence of *PbCAMTA3* and *PbTIC55* between the European and Asian pear populations. **A**, **B** Genotypes of “Chr13_5783950” in *PbCAMTA3* (**A**) and “Chr13_5786061” in *PbCAMTA3* (**B**) in cultivated Asian and cultivated European pears showing correlations with relative fruit firmness. **C** The gene structure of *PbCAMTA3* (top) and putative functional amino acid domains in the PbCAMTA3 protein (bottom). **D** Gene structure of *PbTIC55* in Chinese white pear (*Pyrus* × *bretschneideri*). **E** The fruit firmness of seven Asian and six European pear accessions that were used for PCR amplification. **F** Sequence alignments of sequences from the first exon of the *PbTIC55* gene from seven Asian and six European pear accessions showing the 12-bp tandem duplication that is unique to Asian pears. **G** The relative expression levels of *PbTIC55* in nine cultivated Asian pears and six cultivated European pears determined by quantitative real-time PCR analysis (qRT-PCR). **H** Expression profiles of *PbTIC55* during fruit development starting at 15 days after fertilization (DAF) in four cultivated pear accessions, including three Asian pears (“Housi,” “Yali,” “Dangshansuli”) and one European pear (“Starkrimson”). Relative gene expression was determined using reads per kilobase of transcript per million (RPKM) mapped reads values

Two of these genes are particularly notable: first, the pear ortholog of the previously characterized calmodulin-binding protein gene *CAMTA3* (*Pbr035644.2*) had haplotypes that were divergent between Asian and European pears (Figs. 6C and 7A, B); second, the cultivated Asian pears carry the AA allele and have firmer fruits than cultivated European pears, which have the GG allele. The SNP results in a predicted change from asparagine to aspartic in the CAMTA3 protein (Fig. 7C). We also found a 9-bp insertion in the CDS of *CAMTA3* in the Asian pear population that is absent in the European pear population. This indel caused a 3-amino acid insertion (A-G-L: alanine, glycine, and lysine) in the CAMTA3 protein in Asian pears (Additional file 19: Fig. S6). Previous studies in *Arabidopsis* have demonstrated that disruption of *CAMTA3* results in early senescence [17, 31]. In general, fruit firmness slowly decreases during fruit ripening (Additional file 20: Fig. S7). These results indicate that *CAMTA3* might regulate fruit firmness by mediating maturity and senescence in pears.

The second notable gene is *Pbr019967.1* (Fig. 6C), which is located within the aforementioned 3-Mbp IBD-ER that is common to both Asian and European pears. A BLAST search identified *Pbr019967.1* as the likely pear ortholog of *Arabidopsis TIC55*. To further determine whether *TIC55* contributes to the differences in firmness between Asian and European pears, we investigated the gene structure of *TIC55* and its genetic divergence between Asian and European pears. The *PbTIC55* gene contains three exons and two introns (Fig. 7D) and encodes a predicted protein of 575 amino acid residues with a calculated molecular mass of 64.21 kDa and an isoelectric point of 8.89. Alignments of the coding region sequences (CDS) of the *PbTIC55* gene from European and Asian pear accessions revealed the presence of a nucleotide polymorphism: exon 1 of *PbTIC55* from the cultivated European accessions is 12 bp shorter compared to the Asian accessions, and this was confirmed by Sanger sequencing of the gene from seven Asian and six European pears with significant firmness differences (Fig. 7E). The length difference is due to the duplication of a 12-bp sequence within exon 1, and all seven of the Asian pear accessions were found to carry this 12-bp tandem repeat (Fig. 7F) (Additional file 21: Fig. S8).

To further investigate the effect(s) of this 12-bp tandem repeat on *TIC55*, we compared the expression level of *TIC55* between the Asian pear group (nine cultivated varieties) and the European pear group (six cultivated varieties) using quantitative real-time PCR (qRT-PCR) analysis. The results indicated that the expression of *TIC55* is significantly higher in the Asian pear group than in the European pear group (*T*-test, *p* < 0.001) (Fig. 7G). Asian pears are known to have a relatively slower rate of fruit softening compared to European pears. The gene expression results further supported the idea that 12-bp tandem repeat is associated with fruit softening in Asian pear fruits.

In general, fruit firmness slowly decreases during the fruit development process (Additional file 20: Fig. S7). To verify the function of the *TIC55* gene, we investigated the expression pattern of *TIC55* during pear fruit development in both Asian and European pears using transcriptome data. An increase in the expression level of *TIC55* was found during the development of Asian pear fruits, while *TIC55* was expressed at very low levels or was not expressed at all during the development of European pear fruits (Fig. 7H). We next selected two pear accessions to quantify the relative expression level of *TIC55* during fruit development using qRT-PCR analysis (Additional file 22: Fig. S9). The qRT-PCR results were consistent with the results of transcriptome data analysis. In general, fruit firmness slowly decreases during fruit development, and European pears soften faster than Asian pears. These results indicated that *TIC55* expression acts to repress fruit softening.

## Discussion

We examined the genomes of 113 pear accessions to estimate the demographic history and identify the landscape of gene flow during the independent domestications of Asian and European pears. A 3-Mbp IBD-ER showed strong selective signals that occurred during the domestication of both Asian and European pears, and this IBD-ER harbors the *TIC55* locus, which is significantly associated with fruit softening. We identified a 12-bp insertion mutation in the first exon of *TIC55* in Asian pear accessions that potentially explains the differences in fruit softening between Asian and European pears. This study provides novel insights into our understanding of the genetic basis of fruit softening during pear domestication. Identification of genes associated with fruit firmness will facilitate future molecular studies and will also aid in the genetic improvement and breeding efforts for pears.

In maize, it has been reported that the lengths of IBD tracts range between 3 and 4 Mbp [8, 32]. However, in this study, we found that most of the IBD tracts were between 1500 and 3500 bp in length, which is substantially shorter than the IBD tracts detected in maize. This finding may be attributed to self-incompatibility in pears, because a higher incidence of genetic recombination can break up long IBD tracts [6]. Compared to IBD-ERs, chromosomal segments lacking obvious IBD regions exhibit higher genetic diversity and typically undergo relatively weaker selective pressure [8]. In pear, the *S*-locus for self-incompatibility is located at the end of chromosome 17 and shows a high level of diversity and a rapid evolution rate [3]. In our study, we found no shared IBD tracts at the *S*-locus between wild and cultivated pears (*X*^2^, *p*-value < 2.2e − 16). This finding is consistent with the observations in maize showing that the genomic regions near the known cross-incompatibility loci are likely to be particularly resistant to gene flow [33].

Hormone levels are an important factor that is associated with fruit firmness and fruit size. In our study, the KEGG pathway analysis of genes located in the 3-Mb IBD-ER indicated that these genes are related to zeatin biosynthesis. Zeatin is a natural cytokinin hormone that is produced in plants [34]. Cytokinin levels are decreased during fruit ripening, and elevated levels of cytokinin contribute to delayed ripening [35, 36]. During the fruit ripening process, ethylene is involved in a range of physiological and biochemical changes including chlorophyll degradation, fruit softening, and the production of volatiles [37]. The effect of ethylene on fruit firmness is mainly reflected in ethylene’s effect on pectin metabolism enzymes in the cell wall [38, 39]. In addition, Xin et al. reported that ethylene is required for cucumber fruit elongation, which further affects cucumber fruit size [40]. ABA regulates fruit development by interacting with other hormones. For example, ABA induces cell enlargement during tomato fruit growth by decreasing the synthesis of ethylene, which further influences fruit size in tomatoes [41]. A decade of studies have shown that ABA is an important promotor of fruit softening that mediates transcriptional changes in genes that encode enzymes related to cell wall degradation [42, 43]. For example, the expression level of *FaRGlyase1*, a gene that encodes the enzyme rhamnogalacturonate lyase, was shown to be positively regulated by ABA and negatively regulated by auxins [44].

Fruit firmness is a critical quality trait because it determines the quality grading of fruit and strongly affects shelf life [45]. Fruits of cultivated pears are less firm than fruits of wild pear relatives, although it is important to note that excessive fruit softening is deleterious for long-term storage. While firmness has been widely investigated in many fruits, including sweet cherry, strawberry, and peach [46–49], knowledge of the genetic basis of the differential fruit firmness in pears is rather limited. The process of fruit ripening is usually accompanied by fruit softening (Additional file 20: Fig. S7) [50, 51]. It has been determined that *CAMTA3* plays a critical role in ethylene signaling by directly regulating the expression of both *NON-RACE-SPECIFIC DISEASE RESISTANCE1* (*NDR1*) and *EIN3* in *Arabidopsis* [17, 31]. In this study, we have identified another gene, *TIC55*, that is found within a 3-Mbp IBD-ER in the pear genome that has undergone strong positive selection during domestication. Previous studies reported that *TIC55* functions in chlorophyll breakdown during leaf senescence and fruit ripening [52, 53].

**Fig. 8:**
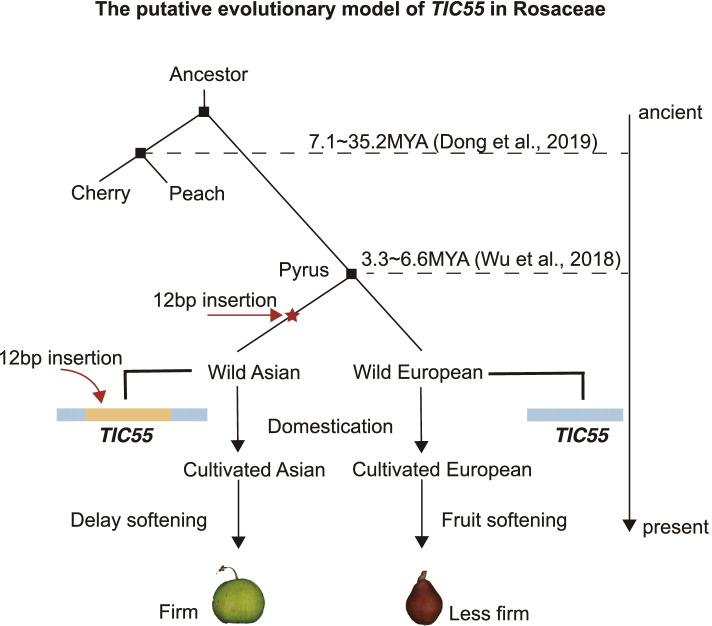
A putative evolutionary model for the *TIC55* gene in *Pyrus*. The tandem duplication that includes the 12-bp insertion in *TIC55* is only present in Asian pear accessions. Compared to European pears, peaches, and sweet cherries, Asian pears are known to have a relatively slower rate of fruit softening accompanied by a relatively long storage life. Thus, it is likely that the 12-bp insertion in *TIC55* contributes to delayed fruit softening

Asian and European pears display extensive phenotypic differences, such as their stone cell content, fruit firmness, and flavor [3], and these differences reflect their independent domestications. Of these traits, probably the most apparent trait difference between these two populations is that of fruit firmness. Compared to European pears, the fruits of Asian pears are significantly firmer, yet the underlying genetic mechanisms contributing to this trait are unclear. Based on genotypic divergence and PCR amplification verification, we have found a 12-bp insertion mutation in the first exon of the *TIC55* gene that is unique to Asian pears. This mutation is likely to be involved in the observed differences in fruit softening phenotypes and could potentially explain the differences in the trait between Asian and European pears (Fig. 8).

## Conclusions

The objectives of our study were to assess the identity-by-descent (IBD) landscape of 113 diverse pear accessions to investigate how historical gene flow has shaped fruit traits in Asian and European pears. A total of 3,320,796 IBD tracts were identified in pairwise comparisons between the 113 pear accessions. Notably, a 3-Mbp region that is shared between Asian IBD-enriched regions (IBD-ERs) and European IBD-ERs was identified on chromosome 15, and both XP-CLR and *F*_*ST*_ analysis strongly supported the hypothesis that the 3-Mbp IBD-ERs had experienced selection during pear domestication. One fruit firmness-associated gene, *TIC55*, was found to be located within the 3-Mbp IBD-ER that is shared between Asian and European pears. Furthermore, we identified a tandem repeat that results from a 12-bp duplication mutation found in the first exon of *TIC55* that is unique to Asian pears, which have a relatively slower rate of fruit softening than European pears. This study investigated the historical gene flow and highlighted its phenotypic effects on pear fruit softening, with important implications for understanding the independent domestications of Asian and European pears.

## Methods

### Acquisition of genome sequences

We used genomic resequencing data for 113 pear accessions that represent the five major cultivated pear groups and the most important wild *Pyrus* species in the world that was generated in our previous study. The resequenced pear accessions included 63 Asian (31 cultivated and 32 wild) and 50 European (25 cultivated and 25 wild) accessions [3].

### SNP source and pairwise IBD detection

SNP calling was performed following previously described protocols [3]. A total of 18,302,883 SNPs were identified in all 113 accession genomes, and these were used as inputs for IBD identification. First, all 113 accessions were phased in Beagle (version 4.0). Second, the IBD tracts were extracted with the Beagle IBD function, as described by Browning and Browning [54], with the LOD score set to > 3.0. To profile the frequency of IBD tracts along each chromosome, the chromosomes were divided into 500-kb windows, and the numbers of detected IBD tracts for each group in each window were counted. These results were illustrated using the Circos tool (version 0.69) [55].

### *FST* analysis, XP-CLR analysis, and XP-EHH analysis

The package VCFtools (version 0.1.17) was used to compute pairwise *F*_*ST*_ values between the wild and cultivated pear populations [56]. The XP-CLR software was used to estimate XP-CLR values between the wild and cultivated populations [25]. The regions harboring XP-CLR values in the top 20% (Asian: XP-CLR value ≥ 5.17; European, XP-CLR value ≥ 2.33) were deemed to be the selection domestication region. The *F*_*ST*_ values and XP-CLR values were calculated using a sliding window of 500 kb and a step length of 50 kb. The regions harboring *F*_*ST*_ values > 1.5 were considered to be selection domestication regions. We also performed comparisons between the wild and cultivated populations in both Asian and European pears using cross-population extended haplotype homozygosity (XP-EHH). For XP-EHH analysis, we first phased and imputed genotyping data using the Beagle software. We then used the Selscan software to measure the XP-EHH scores with the default parameters. The top 1% of XP-EHH signals were considered to be selection signals.

### Genotyping using a 200 K AXIOM® PyrSNP array

A total of 164 diverse pear accessions were selected and genotyped using the 200 K AXIOM® PyrSNP array as previously described [30]. Twelve of the pear accessions were shared between the 113 resequenced pear accessions and the 164 accessions used in the GWAS in this study. A kit (Tiangen Biotech Co., Ltd., Beijing, China) was used to extract genomic DNA from frozen fruit tissues from each of the 164 pear accessions. All raw hybridization intensity data were analyzed using the Axiom Analysis Suite (v.1.1.1) with a diploid threshold configuration (Affymetrix, Santa Clara, CA). Samples with call rates (CR) > 97 and dish quality control (DQC) values > 0.82 were retained and subjected to further analysis.

Based on the reliability of the SNPs, SNPs from the results obtained from the Axiom Analysis Suite were classified into the following six categories using the “SNPolisher” R package: “PolyHighResolution” (PHR), “NoMinorHom” (NMH), “OTV,” “MonoHighResolution,” “CallRateBelowThreshold,” and “other.” Those SNPs classified as “other” had the lowest quality and were excluded from further analysis.

### A genome-wide association study (GWAS) for pear fruit firmness

The pear fruit firmness trait was measured using a Brookfield CT3 Texture Analyzer (kg/cm^2^) (AMETEK Brookfield, Middleboro, MA). Three pear accessions were randomly selected as three biological replicates. Three surface points along each pear fruit were randomly selected to measure fruit firmness, and the average value of these measurements was designated as the fruit firmness of each pear fruit analyzed.

The Genomic Association and Prediction Integrated Tool (GAPIT) package was used to conduct GWAS using the mixed linear model (MLM) [57]. The GWAS significance threshold was set to 3.41 × 10^−5^ (1/*n*, where *n* is the number of SNPs used in GWAS). QQman (R package) was used to create Manhattan plots [58].

Linkage disequilibrium (LD) analyses were performed to search for candidate genes in regions 50 kb upstream and 50 kb downstream of significant SNPs using plink (v1.07) [59]. LDheatmap (R package) was used to display linkage disequilibrium values of SNPs within a 100-kb candidate region [60].

### Quantitative real-time PCR analysis (qRT-PCR)

We selected nine Asian and seven European pear accessions in which to assay the expression pattern of *PbTIC55* using qRT-PCR. The Asian pear group comprised the cultivars “Hongxiao,” “Oushuu,” “Saigyoku,” “Qiuxiangshui,” “Hehuali,” “Mansoo,” “Lelingshali,” “Balengli,” and “Meirensu.” The European pear group included the cultivars “La France,” “Jana,” “Bartlett,” “Bosco,” “Clarke,” and “Comice.”

Total RNA was extracted using the Plant Total RNA isolation Plus kit (Foregene; http://www.foregene.com), and first-strand cDNA was synthesized using the EasyScript® First-Strand cDNA Synthesis SuperMix Kit (Transgene; https://www.transgen.com.cn/rt_pcr.html). Primers were designed to amplify genes using online software available from NCBI (National Center for Biotechnology Information) (https://www.ncbi.nlm.nih.gov/tools/primerblast/) (Additional file 13: Table S13). As described previously [61], LightCycler 480 SYBR GREEN I Master (Roche) was used for the qRT-PCR analyses. Each 10 μl reaction contained 150 ng of template cDNA, 0.5 μM of each primer, and 5 μl of LightCycler 480 SYBR GREEN I Master. All reactions were performed in 96-well plates with three replicates for each cDNA sample. The qRT-PCR amplification conditions were as follows: 3 min at 95 °C followed by 45 cycles of 95 °C for 3 s, 60 °C for 10 s, and 72 °C for 30 s. Fluorescence signal data was collected at 60 °C. The *Pyrus TUB* gene was used as the internal control. The average threshold cycle (Ct) of each cDNA sample was calculated. Relative gene expression levels were calculated using the 2^−ΔΔCt^ method as described [62].

### KEGG analysis

A Kyoto Encyclopedia of Genes and Genomes (KEGG) pathway analysis was performed using KOBAS 3.0 [63].

### Statistical analysis

All statistical tests were performed using R language basic packages (version 3.5.1) (https://www.R-project.org/). 

## Supplementary Information

**Additional file 1:** **Table S1.** Fruit firmness in wild and cultivated pears.

**Additional file 2:** **Table S2.** Whole genome sequencing (WGS) data for 113 pear accessions used in our study.

**Additional file 3:** **Table S3.** Length distribution of putative IBD tracts between populations and within populations. WE, wild European; CA, cultivated Asian; WE, wild European; CE, cultivated European. Bin size = 500 bp.

**Additional file 4:** **Table S4.** Numbers of IBD tracts in pear genomes (500 kb window) in 10 comparisons.

**Additional file 5:** **Table S5.** The IBD-enriched regions (IBD-ERs) in Asian and European pear populations.

**Additional file 6:** **Table S6.** Genes in IBD-enriched regions (IBD-ERs) identified between wild Asian and cultivated Asian pears.

**Additional file 7:** **Table S7.** Genes in IBD-enriched regions (IBD-ERs) identified between wild European and cultivated European pears.

**Additional file 8:** **Table S8. **The collection sites of 164 pear accessions used in the GWAS in this study.

**Additional file 9:** **Table S9.** Quality control of 200,481 SNPs from the SNP array.

**Additional file 10:** **Table S10.** SNPs, SNP density, and gene density of all 29,269 high-quality SNPs on all 17 pear chromosomes.

**Additional file 11:** **Table S11.** A summary of the categorized SNPs.

**Additional file 12:** **Table S12.** Two significant SNPs associated with fruit firmness.

**Additional file 13:** **Table S13.** Names and sequences (5’ to 3’) of all oligonucleotide primers used in this study.

**Additional file 14:** **Fig. S1.** Distribution of IBD tract lengths within populations (cultivated Asian-cultivated Asian, wild Asian-wild Asian, cultivated European-cultivated European, and wild European-wild European) and between populations (cultivated Asian-wild Asian, cultivated Asian-cultivated European, cultivated Asian-wild European, wild Asian-cultivated European, wild Asian-wild European, and cultivated European-wild European). WE, wild European; CA, cultivated Asian; WE, wild European; and CE, cultivated European.

**Additional file 15:** **Fig. S2.** Distribution of IBD tracts along the 17 pear chromosomes. (A) Distribution of IBD tracts in the cultivated Asian-wild Asian (CA-WA; red bars) and cultivated European-wild European (CE-WE; blue bars) comparisons. (B) Lengths of all 17 pear chromosomes. (C and D) KEGG functional enrichment of genes from IBD-ERs of Asian (C) and European (D) pears. Pathways related to carbohydrate metabolism are enclosed in red boxes. The sizes of the dots correspond to the number of genes.

**Additional file 16:** **Fig. S3.** KEGG analysis of genes within the 3 Mbp IBD-ER.

**Additional file 17:** **Fig. S4.** The distribution of XP-EHH scores on the 17 pear chromosomes in the WA-CA comparison (A) and WE-CE comparison (B). The cutoff lines represent the top 1% of selection signals (Asian pear population: XP-EHH > 0.828041; European pear population: XP-EHH > 0.680248).

**Additional file 18:** **Fig. S5.** GWAS of fruit firmness. Quantile-Quantile (Q-Q) plots of a genome-wide association analysis for fruit firmness.

**Additional file 19:** **Fig. S6.** Sanger DNA sequencing of the *CAMTA3* gene in Asian and European pears showing the 9-bp insertion mutation unique to Asian pears. (A) Sequence alignment of the coding sequences of the *PbCAMTA3 *gene from eight European and nine Asian pear accessions. (B) Gene structure showing the introns and exons of *PbCAMTA3 *(upper) and the predicted domains (IPT (transcription factor immunoglobulin), IQ motifs (calmodulin-binding), CG-1 (a DNA-binding domain specific to sequence), and ankyrin (ANK) repeats) in the PbCAMTA3 protein structure.

**Additional file 20:** **Fig. S7.** Fruit firmness of four pear accessions, including ‘Hosui’ (cultivated Asian), ‘Nanguoli’ (cultivated Asian), ‘Starkrimson’ (cultivated European), and ‘Yali’ (cultivated Asian) during five developmental stages of fruit growth. S1 corresponds to the physiological fruit drop stage at 30 days after flowering (DAF), S2 corresponds to the rapid fruit enlargement stage at 55 DAF, S3 corresponds to the fruiting stage at 85 DAF, S4 corresponds to the pre-maturity fruit stage at 115 DAF, and S5 corresponds to the mature fruit stage.

**Additional file 21:** **Fig. S8.** Distribution of sequencing reads that mapped to the promoter region (upstream 2,000 bp) of the *PbTIC55* gene from 113 pear accessions derived from four populations (wild Asian, cultivated Asian, wild European, and cultivated European populations). The results of this analysis did not identify any potential indels or SVs in the promoter region of *TIC55* between the Asian and European pear populations.

**Additional file 22:** **Fig. S9.** The relative expression levels of *TIC55* during pear fruit development in ‘Dangshansuli’ (Asian pear) and ‘Starkrimson’ (European pear) based on qRT-PCR. DAF: days after flowering; DAH: days after harvest.

## Data Availability

The pear (*Pyrus bretschneideri*) genome sequences were downloaded from NCBI (BioProject accession: PRJNA157875) [64]. The genomic resequencing data for the 113 pear accessions was obtained from a previous study (BioProject accession: PRJNA381668) [65].

## Abbreviations

IBD: Identity-by-descent
IBD-ERs: IBD-enriched regions
GWAS: Genome-wide association analysis
MLM: Mixed linear model
KEGG: Kyoto Encyclopedia of Genes and Genomes
SNP: Single nucleotide polymorphism
MAF: Minor allele frequency
LD: Linkage disequilibrium
GAPIT: Genomic Association and Prediction Integrated Tool
*CAMTA3*: *Calmodulin Binding Transcription Activator3*
*TIC55*: *Translocon At The Inner Chloroplast Envelope55*

## References

[1] Mirabdulbaghi M (2015). Investigations on determination of nutritional status of pear trees according to a new index - Deviation From Optimum Percentage (DOP). Cercet Agron Mold.

[2] Janick J, Moore JN (1996). Fruit breeding.

[3] Wu J, Wang Y, Xu J, Korban SS, Fei Z, Tao S, Ming R, Tai S, Khan AM, Postman JD (2018). Diversification and independent domestication of Asian and European pears. Genome Biol.

[4] Li J, Zhang M, Li X, Khan A, Kumar S, Allan AC, Lin-Wang K, Espley RV, Wang C, Wang R (2022). Pear genetics: recent advances, new prospects, and a roadmap for the future. Hortic Res.

[5] Ma Y, Wang J, Hu Q, Li J, Sun Y, Zhang L, Abbott RJ, Liu J, Mao K (2019). Ancient introgression drives adaptation to cooler and drier mountain habitats in a cypress species complex. Commun Biol.

[6] Martin SH, Jiggins CD (2017). Interpreting the genomic landscape of introgression. Curr Opin Genet Dev.

[7] Zhao K, Wright M, Kimball J, Eizenga G, McClung A, Kovach M, Tyagi W, Ali ML, Tung CW, Reynolds A (2010). Genomic diversity and introgression in O. sativa reveal the impact of domestication and breeding on the rice genome. PloS One.

[8] Li X, Jian Y, Xie C, Wu J, Xu Y, Zou C (2017). Fast diffusion of domesticated maize to temperate zones. Sci Rep.

[9] Wu J, Wang Z, Shi Z, Shu Z, Zhang S (2013). The genome of the pear (Pyrus bretschneideri Rehd.). Genome Res.

[10] Julian JD, Zabotina OA (2022). Xyloglucan biosynthesis: from genes to proteins and their functions. Front Plant Sci.

[11] Bird CR, Smith CJS, Ray JA, Moureau P, Bevan MW, Bird AS, Hughes S, Morris PC, Grierson D, Schuch W (1988). The tomato polygalacturonase gene and ripening-specific expression in transgenic plants. Plant Mol Biol.

[12] Sanchez N, Gutiérrez-López GF, Cáez-Ramírez G (2020). Correlation among PME activity, viscoelastic, and structural parameters for Carica papaya edible tissue along ripening. J Food Sci.

[13] Smith DL, Abbott JA, Gross KC (2002). Down-regulation of tomato β-galactosidase 4 results in decreased fruit softening. Plant Physiol.

[14] Uluisik S, Chapman NH, Smith R, Poole M, Adams G, Gillis RB, Besong TMD, Sheldon J, Stiegelmeyer S, Perez L (2016). Genetic improvement of tomato by targeted control of fruit softening. Nat Biotechnol.

[15] Harada T, Sunako T, Wakasa Y, Soejima J, Satoh T, Niizeki M (2000). An allele of the 1-aminocyclopropane-1-carboxylate synthase gene (Md-ACS1) accounts for the low level of ethylene production in climacteric fruits of some apple cultivars. Theor Appl Genet.

[16] Costa F, Stella S, Van de Weg WE, Guerra W, Cecchinel M, Dallavia J, Koller B, Sansavini S (2005). Role of the genes Md-ACO1 and Md-ACS1 in ethylene production and shelf life of apple (Malus domestica Borkh). Euphytica.

[17] Nie H, Zhao C, Wu G, Wu Y, Chen Y, Tang D (2012). SR1, a calmodulin-binding transcription factor, modulates plant defense and ethylene-induced senescence by directly regulating NDR1 and EIN3. Plant Physiol.

[18] Zhang Z, Zhang H, Quan R, Wang X-C, Huang R (2009). Transcriptional regulation of the ethylene response factor LeERF2 in the expression of ethylene biosynthesis genes controls ethylene production in tomato and tobacco. Plant Physiol.

[19] Xiao Y-y, Chen J-y, Kuang J-f (2013). Shan W, Xie H, Jiang Y-m, Lu W-j: Banana ethylene response factors are involved in fruit ripening through their interactions with ethylene biosynthesis genes. J Exp Bot.

[20] Hu Y, Han Z, Sun Y, Wang S, Wang T, Wang Y, Xu K, Zhang X, Xu X, Han Z (2020). ERF4 affects fruit firmness through TPL4 by reducing ethylene production. Plant J.

[21] Kuai B, Chen J, Hörtensteiner S (2017). The biochemistry and molecular biology of chlorophyll breakdown. J Exp Bot.

[22] Wang X, Chen L, Ma J (2019). Genomic introgression through interspecific hybridization counteracts genetic bottleneck during soybean domestication. Genome Biol.

[23] Saito T (2016). Advances in Japanese pear breeding in Japan. Breeding Sci.

[24] Yamamoto T, Terakami S, Moriya S, Hosaka F, Kurita K, Kanamori H, Katayose Y, Saito T, Nishitani C (2013). DNA markers developed from genome sequencing analysis in Japanese pear (Pyrus pyrifolia). Acta Hortic.

[25] Chen H, Patterson N, Reich D (2010). Population differentiation as a test for selective sweeps. Genome Res.

[26] Wang L, He F, Huang Y, He J, Yang S, Zeng J, Deng C, Jiang X, Fang Y, Wen S (2018). Genome of wild mandarin and domestication history of mandarin. Mol Plant.

[27] Paterson AH, Lin Y-R, Li Z, Schertz KF, Doebley JF, Pinson SRM, Liu S-C, Stansel JW, Irvine JE (1995). Convergent domestication of cereal crops by independent mutations at corresponding genetic loci. Science.

[28] Lenser T, Theißen G (2013). Molecular mechanisms involved in convergent crop domestication. Trends Plant Sci.

[29] Cheng F, Sun R, Hou X, Zheng H, Zhang F, Zhang Y, Liu B, Liang J, Zhuang M, Liu Y (2016). Subgenome parallel selection is associated with morphotype diversification and convergent crop domestication in Brassica rapa and Brassica oleracea. Nat Genet.

[30] Li X, Singh J, Qin M, Li S, Zhang X, Zhang M, Khan A, Zhang S, Wu J (2019). Development of an integrated 200K SNP genotyping array and application for genetic mapping, genome assembly improvement and genome wide association studies in pear (Pyrus). Plant Biotechnol J.

[31] Consonni C, Humphry ME, Hartmann HA, Livaja M, Durner J, Westphal L, Vogel J, Lipka V, Kemmerling B, Schulze-Lefert P (2006). Conserved requirement for a plant host cell protein in powdery mildew pathogenesis. Nat Genet.

[32] Zhou Z, Zhang C, Zhou Y, Hao Z, Wang Z, Zeng X, Di H, Li M, Zhang D, Yong H (2016). Genetic dissection of maize plant architecture with an ultra-high density bin map based on recombinant inbred lines. BMC Genomics.

[33] Hufford MB, Lubinksy P, Pyhäjärvi T, Devengenzo MT, Ellstrand NC, Ross-Ibarra J (2013). The genomic signature of crop-wild introgression in maize. PLoS Genet.

[34] Martin RC, Mok MC, Mok DWS (1999). A gene encoding the cytokinin enzyme zeatinO-xylosyltransferase of Phaseolus vulgaris1. Plant Physiol.

[35] McAtee P, Karim S, Schaffer R, David K (2013). A dynamic interplay between phytohormones is required for fruit development, maturation, and ripening. Front Plant Sci.

[36] Kumar R, Khurana A, Sharma AK (2014). Role of plant hormones and their interplay in development and ripening of fleshy fruits. J Exp Bot.

[37] Aubert C, Chanforan C (2007). Postharvest changes in physicochemical properties and volatile constituents of apricot (Prunus armeniaca L.). Characterization of 28 cultivars. J Agr Food Chem.

[38] Merchante C, Vallarino JG, Osorio S, Aragüez I, Villarreal N, Ariza MT, Martínez GA, Medina-Escobar N, Civello MP, Fernie AR (2013). Ethylene is involved in strawberry fruit ripening in an organ-specific manner. J Agr Food Chem.

[39] Villarreal NM, Marina M, Nardi CF, Civello PM, Martínez GA (2016). Novel insights of ethylene role in strawberry cell wall metabolism. Plant Sci.

[40] Xin T, Zhang Z, Li S, Zhang S, Li Q, Zhang Z-H, Huang S, Yang X (2019). Genetic regulation of ethylene dosage for cucumber fruit elongation. Plant Cell.

[41] Nitsch L, Kohlen W, Oplaat C, Charnikhova T, Cristescu S, Michieli P, Wolters-Arts M, Bouwmeester H, Mariani C, Vriezen WH (2012). ABA-deficiency results in reduced plant and fruit size in tomato. J Plant Physiol.

[42] Moya-León MA, Mattus-Araya E, Herrera R (2019). Molecular events occurring during softening of strawberry fruit. Front Plant Sci.

[43] Lindo-García V, Muñoz P, Larrigaudière C, Munné-Bosch S, Giné-Bordonaba J (2020). Interplay between hormones and assimilates during pear development and ripening and its relationship with the fruit postharvest behaviour. Plant Sci.

[44] Molina-Hidalgo FJ, Franco AR, Villatoro C, Medina-Puche L, Mercado JA, Hidalgo MA, Monfort A, Caballero JL, Muñoz-Blanco J, Blanco-Portales R (2013). The strawberry (Fragaria×ananassa) fruit-specific rhamnogalacturonate lyase 1 (FaRGLyase1) gene encodes an enzyme involved in the degradation of cell-wall middle lamellae. J Exp Bot.

[45] Yang L, Huang W, Xiong F, Xian Z, Su D, Ren M, Li Z (2017). Silencing of SlPL, which encodes a pectate lyase in tomato, confers enhanced fruit firmness, prolonged shelf-life and reduced susceptibility to grey mould. Plant Biotechnol J.

[46] Calle A, Balas F, Cai L, Iezzoni A, López-Corrales M, Serradilla MJ, Wünsch A (2020). Fruit size and firmness QTL alleles of breeding interest identified in a sweet cherry ‘Ambrunés’ × ‘Sweetheart’ population. Mol Breeding.

[47] Ornelas-Paz JDJ, Quintana-Gallegos BM, Escalante-Minakata P, Reyes-Hernández J, Ruiz-Cruz S (2017). Relationship between the firmness of Golden Delicious apples and the physicochemical characteristics of the fruits and their pectin during development and ripening. J Food Sci Tech.

[48] Küçükönder H, Vursavuş KK, Üçkardeş F (2014). Determining the factors affecting fruit hardness of different peach types with meta analysis. Turkish J Agric Food Sci Technol.

[49] Døving A, Måge F (2002). Methods of testing strawberry fruit firmness. Acta Agric Scand B Soil Plant Sci.

[50] Valero C, Crisosto CH, Slaughter D (2007). Relationship between nondestructive firmness measurements and commercially important ripening fruit stages for peaches, nectarines and plums. Postharvest Biol Tec.

[51] Brummell DA (2006). Cell wall disassembly in ripening fruit. Funct Plant Biol.

[52] Hauenstein M, Christ B, Das A, Aubry S, Hörtensteiner S (2016). A role for TIC55 as a hydroxylase of phyllobilins, the products of chlorophyll breakdown during plant senescence. Plant Cell.

[53] Chou M-L, Liao W-Y, Wei W-C (2018). Li AY-S, Chu C-Y, Wu C-L, Liu C-L, Fu T-H, Lin L-F: The direct involvement of dark-induced Tic55 protein in chlorophyll catabolism and its indirect role in the MYB108-NAC signaling pathway during leaf senescence in Arabidopsis thaliana. Int J Mol Sci.

[54] Browning SR, Browning BL (2007). Rapid and accurate haplotype phasing and missing-data inference for whole-genome association studies by use of localized haplotype clustering. Am J Hum Genet.

[55] Krzywinski M, Schein J, Birol I, Connors J, Gascoyne R, Horsman D, Jones SJ, Marra MA (2009). Circos: an information aesthetic for comparative genomics. Genome Res.

[56] Danecek P, Auton A, Abecasis G, Albers CA, Banks E, DePristo MA, Handsaker RE, Lunter G, Marth GT, Sherry ST (2011). The variant call format and VCFtools. Bioinformatics.

[57] Lipka AE, Tian F, Wang Q, Peiffer J, Li M, Bradbury PJ, Gore MA, Buckler ES, Zhang Z (2012). GAPIT: genome association and prediction integrated tool. Bioinformatics.

[58] Turner SD. qqman: an R package for visualizing GWAS results using Q-Q and manhattan plots. J Open Source Softw. 2018;3(25):731.

[59] Purcell S, Neale B, Todd-Brown K, Thomas L, Ferreira MAR, Bender D, Maller J, Sklar P, Bakker PIWD, Daly MJ (2007). PLINK: a tool set for whole-genome association and population-based linkage analyses. Am J Hum Genet.

[60] Shin J-H, Blay S, McNeney B, Graham J (2006). LDheatmap: an R function for graphical display of pairwise linkage disequilibria between single nucleotide polymorphisms. J Stat Softw.

[61] Li X, Xue C, Li J, Qiao X, Li L, Yu L, Huang Y, Wu J (2016). Genome-wide identification, evolution and functional divergence of MYB transcription factors in Chinese white pear (Pyrus bretschneideri). Plant Cell Physiol.

[62] Livak KJ, Schmittgen TD (2001). Analysis of relative gene expression data using real-time quantitative PCR and the 2^−ΔΔCT^ method. Methods.

[63] Bu D, Luo H, Huo P, Wang Z, Zhang S, He Z, Wu Y, Zhao L, Liu J, Guo J et al. KOBAS-i: intelligent prioritization and exploratory visualization of biological functions for gene enrichment analysis. Nucleic Acids Res. 2021;49(W1):W317-W325. 10.1093/nar/gkab447 PMC8265193 34086934

[64] Wu J (2012). The genome of the pear (Pyrus bretschneideri Rehd.). NCBI accession: PRJNA157875.

[65] Wu J (2018). Diversification and independent domestication of Asian and European pears. NCBI accession: PRJNA381668.

