# Supplementary figures and images for: An identical-by-descent segment harbors a 12-bp insertion determining fruit softening during domestication and speciation in Pyrus
Source: BMC Biol. 2022 Oct 1;20:215. doi: 10.1186/s12915-022-01409-w (PMC9526952; doi:10.1186/s12915-022-01409-w)

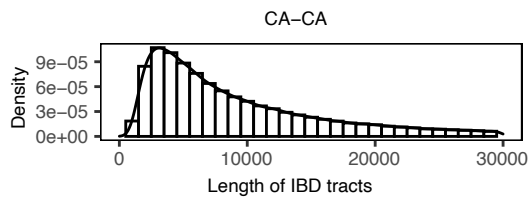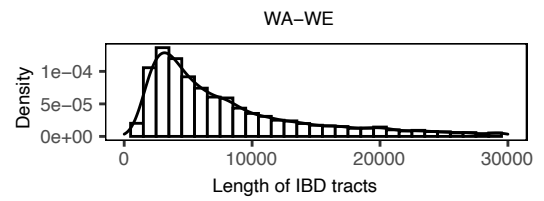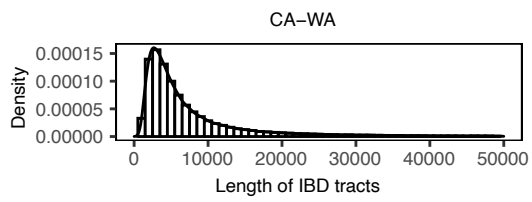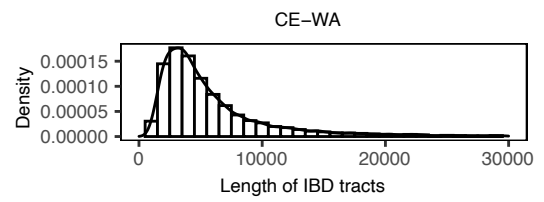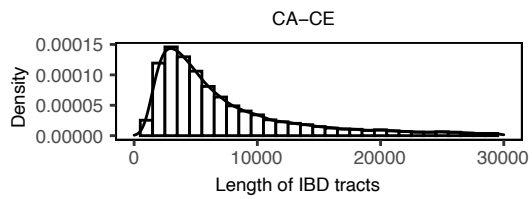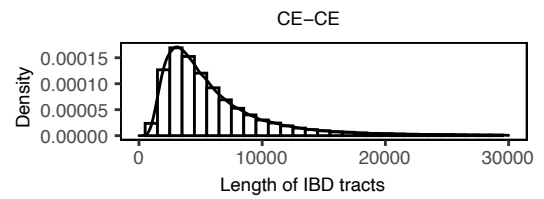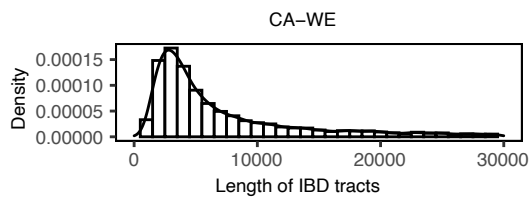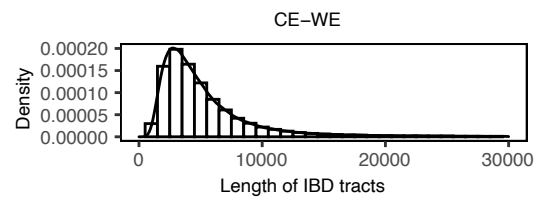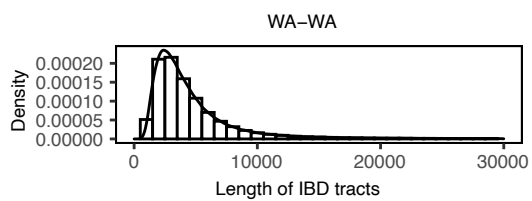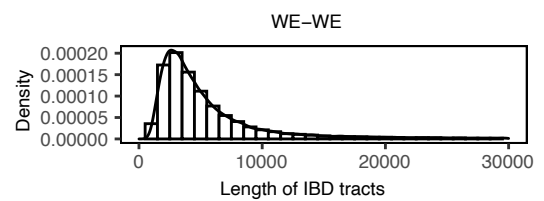

Supplement: Supplementary file 14 — Additional file 14: Fig. S1. Distribution of IBD tract lengths within populations (cultivated Asian-cultivated Asian, wild Asian-wild Asian, cultivated European-cultivated European, and wild European-wild European) and between populations (cultivated Asian-wild Asian, cultivated Asian-cultivated European, cultivated Asian-wild European, wild Asian-cultivated European, wild Asian-wild European, and cultivated European-wild European). WE, wild European; CA, cultivated Asian; WE, wild European; and CE, cultivated European. [file 12915_2022_1409_MOESM14_ESM.pdf]

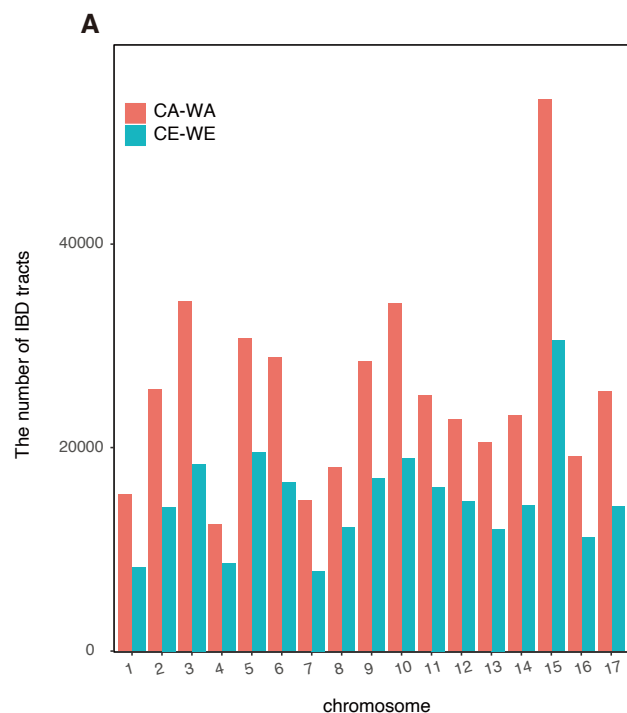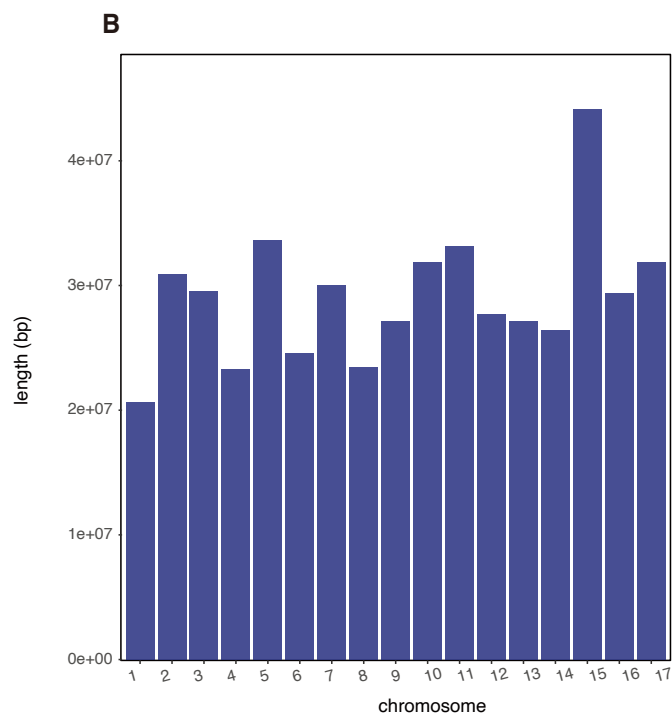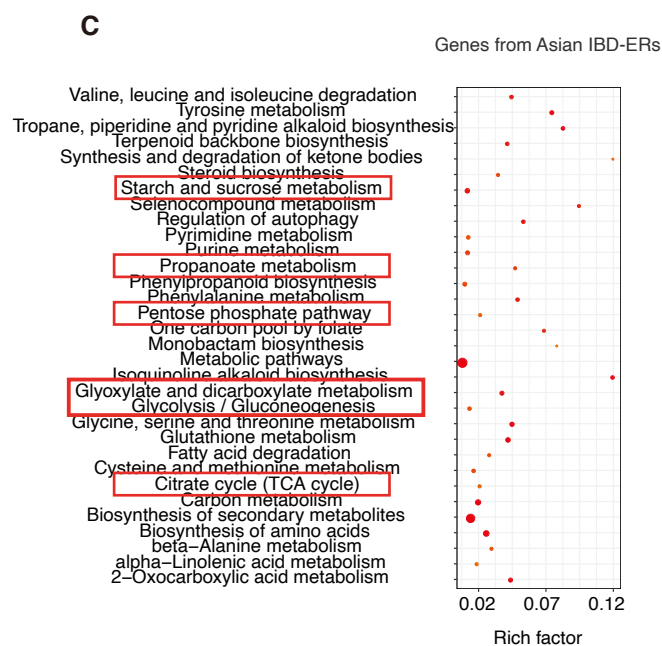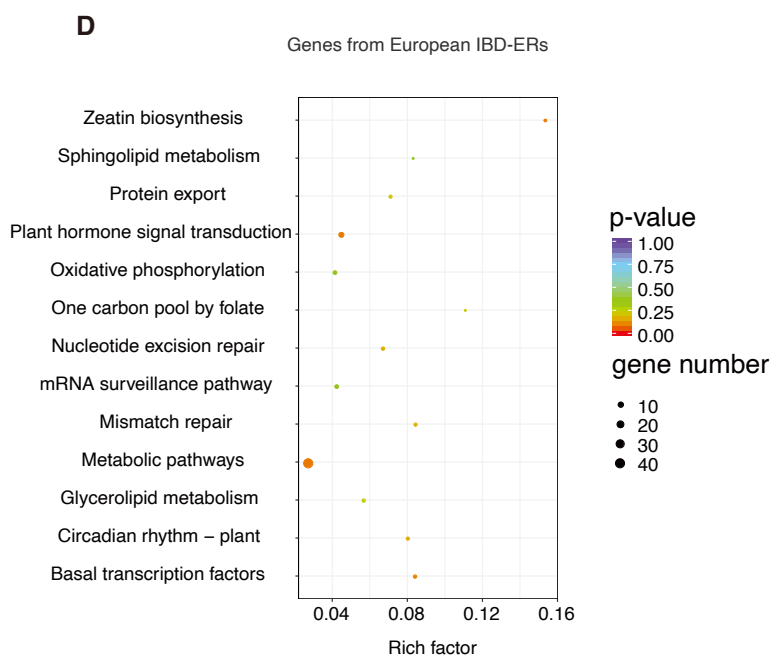

Supplement: Supplementary file 15 — Additional file 15: Fig. S2. Distribution of IBD tracts along the 17 pear chromosomes. (A) Distribution of IBD tracts in the cultivated Asian-wild Asian (CA-WA; red bars) and cultivated European-wild European (CE-WE; blue bars) comparisons. (B) Lengths of all 17 pear chromosomes. (C and D) KEGG functional enrichment of genes from IBD-ERs of Asian (C) and European (D) pears. Pathways related to carbohydrate metabolism are enclosed in red boxes. The sizes of the dots correspond to the number of genes. [file 12915_2022_1409_MOESM15_ESM.pdf]

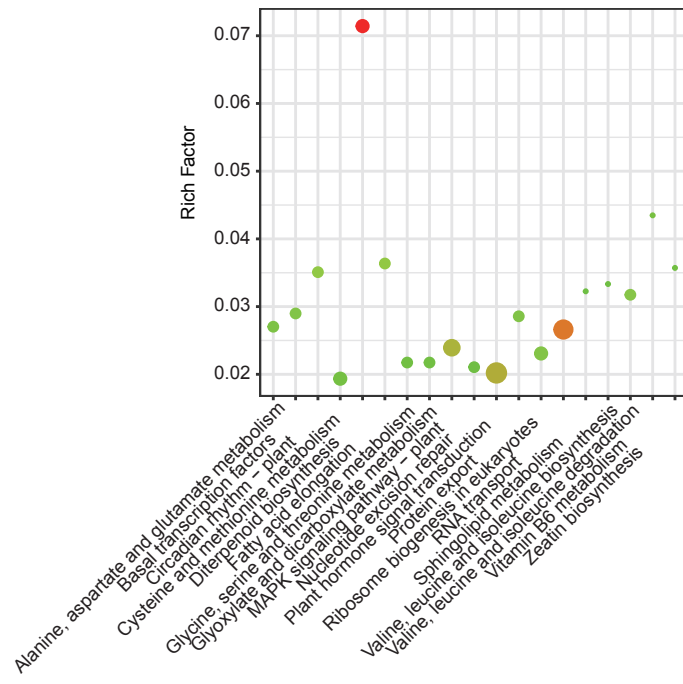

Supplement: Supplementary file 16 — Additional file 16: Fig. S3. KEGG analysis of genes within the 3 Mbp IBD-ER. [file 12915_2022_1409_MOESM16_ESM.pdf]

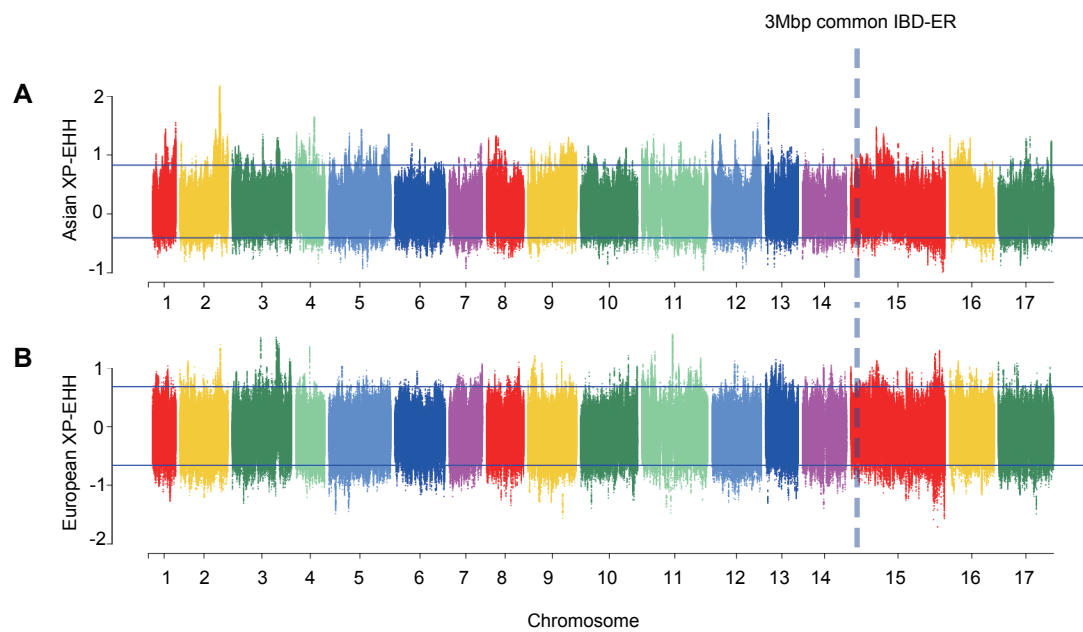

Supplement: Supplementary file 17 — Additional file 17: Fig. S4. The distribution of XP-EHH scores on the 17 pear chromosomes in the WA-CA comparison (A) and WE-CE comparison (B). The cutoff lines represent the top 1% of selection signals (Asian pear population: XP-EHH > 0.828041; European pear population: XP-EHH > 0.680248). [file 12915_2022_1409_MOESM17_ESM.pdf]

Q-Q plot of GWAS p-values

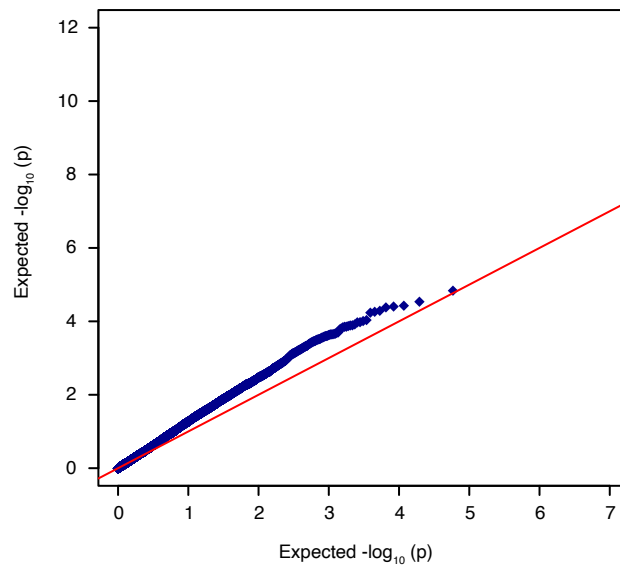

Supplement: Supplementary file 18 — Additional file 18: Fig. S5. GWAS of fruit firmness. Quantile-Quantile (Q-Q) plots of a genome-wide association analysis for fruit firmness. [file 12915_2022_1409_MOESM18_ESM.pdf]

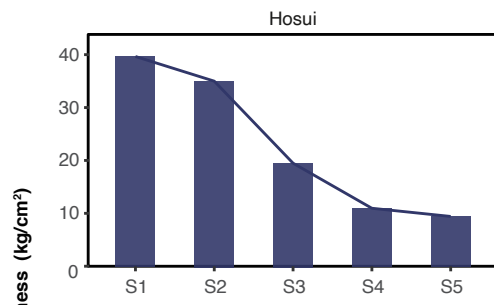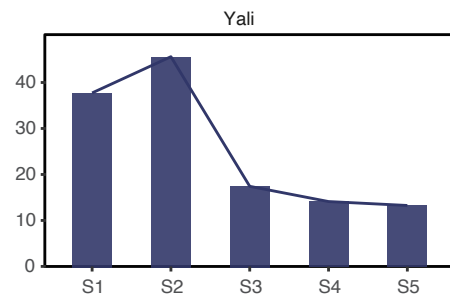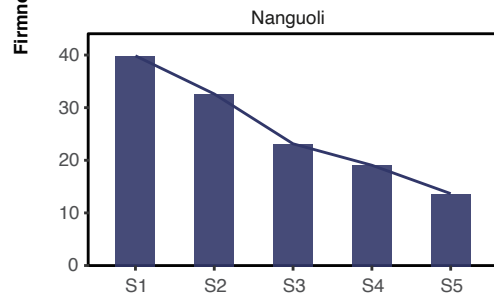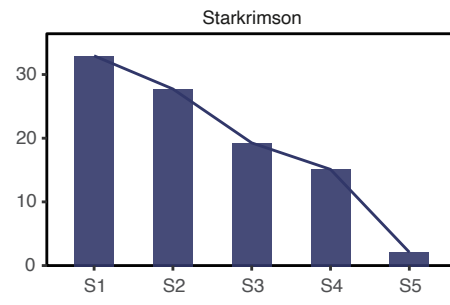

Supplement: Supplementary file 20 — Additional file 20: Fig. S7. Fruit firmness of four pear accessions, including ‘Hosui’ (cultivated Asian), ‘Nanguoli’ (cultivated Asian), ‘Starkrimson’ (cultivated European), and ‘Yali’ (cultivated Asian) during five developmental stages of fruit growth. S1 corresponds to the physiological fruit drop stage at 30 days after flowering (DAF), S2 corresponds to the rapid fruit enlargement stage at 55 DAF, S3 corresponds to the fruiting stage at 85 DAF, S4 corresponds to the pre-maturity fruit stage at 115 DAF, and S5 corresponds to the mature fruit stage. [file 12915_2022_1409_MOESM20_ESM.pdf]

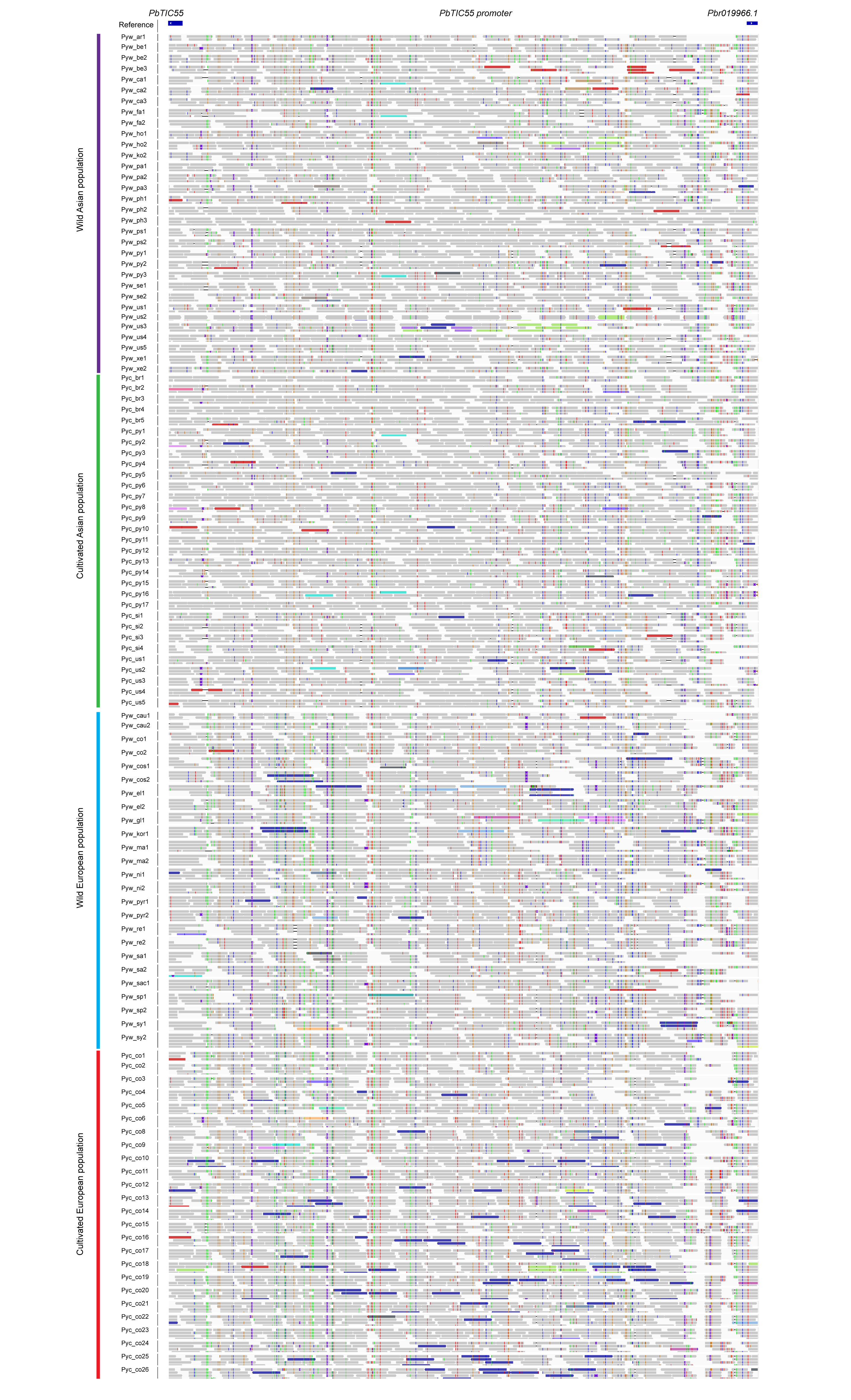

Supplement: Supplementary file 21 — Additional file 21: Fig. S8. Distribution of sequencing reads that mapped to the promoter region (upstream 2,000 bp) of the PbTIC55 gene from 113 pear accessions derived from four populations (wild Asian, cultivated Asian, wild European, and cultivated European populations). The results of this analysis did not identify any potential indels or SVs in the promoter region of TIC55 between the Asian and European pear populations. [file 12915_2022_1409_MOESM21_ESM.png]

Dangshansuli  
(*P.bretschneideri*)

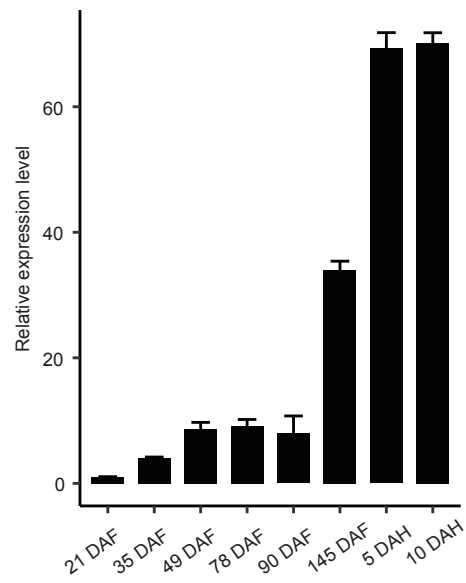

Starkrimson  
(*P.communis*)

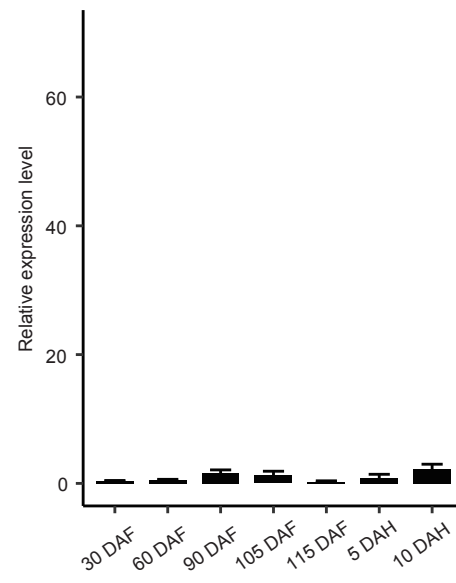

Supplement: Supplementary file 22 — Additional file 22: Fig. S9. The relative expression levels of TIC55 during pear fruit development in ‘Dangshansuli’ (Asian pear) and ‘Starkrimson’ (European pear) based on qRT-PCR. DAF: days after flowering; DAH: days after harvest. [file 12915_2022_1409_MOESM22_ESM.pdf]
