# Supplementary material for: An identical-by-descent segment harbors a 12-bp insertion determining fruit softening during domestication and speciation in Pyrus
Source: BMC Biol. 2022 Oct 1;20:215. doi: 10.1186/s12915-022-01409-w (PMC9526952; doi:10.1186/s12915-022-01409-w)
Supplement: Supplementary file 19 — Additional file 19: Fig. S6. Sanger DNA sequencing of the CAMTA3 gene in Asian and European pears showing the 9-bp insertion mutation unique to Asian pears. (A) Sequence alignment of the coding sequences of the PbCAMTA3 gene from eight European and nine Asian pear accessions. (B) Gene structure showing the introns and exons of PbCAMTA3 (upper) and the predicted domains (IPT (transcription factor immunoglobulin), IQ motifs (calmodulin-binding), CG-1 (a DNA-binding domain specific to sequence), and ankyrin (ANK) repeats) in the PbCAMTA3 protein structure. [file 12915_2022_1409_MOESM19_ESM.pdf]

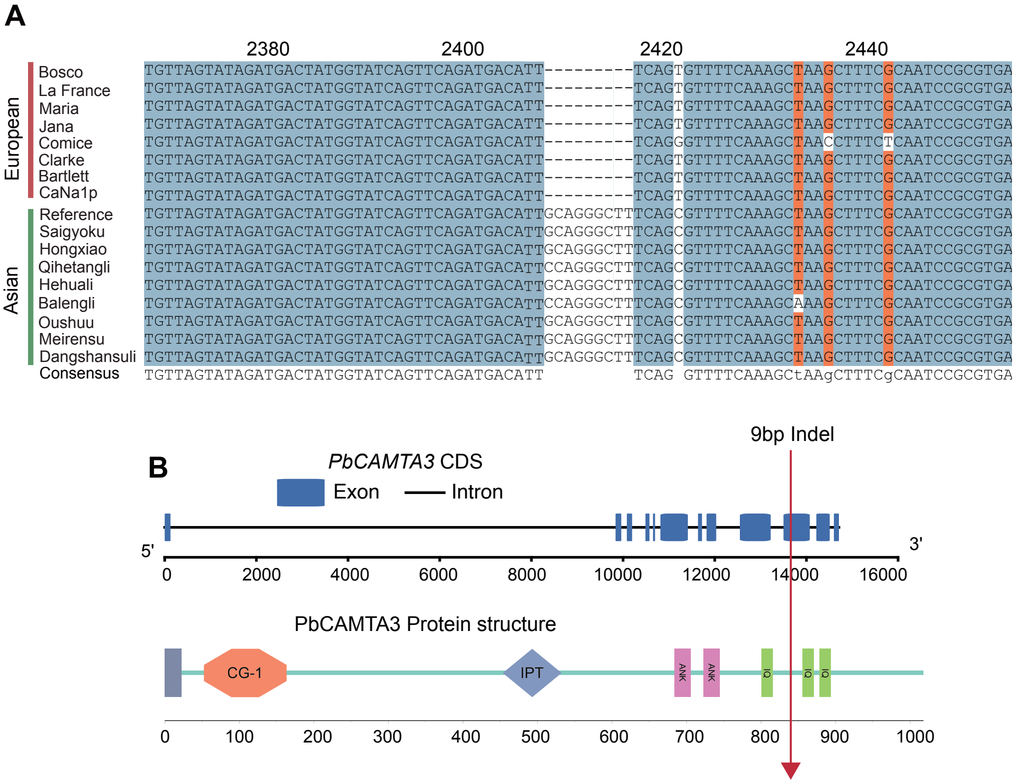

	9bp indel	Amino acid
Hap.A	——	——
Hap.B	GCAGGGCTT	AGL
